# Temporal Dependency and the Structure of Early Looking

**DOI:** 10.1371/journal.pone.0169458

**Published:** 2017-01-11

**Authors:** Daniel S. Messinger, Whitney I. Mattson, James Torrence Todd, Devon N. Gangi, Nicholas D. Myers, Lorraine E. Bahrick

**Affiliations:** 1 Department of Psychology, University of Miami, Coral Gables, Florida, United States of America; 2 Department of Pediatrics, University of Miami, Coral Gables, Florida, United States of America; 3 Department of Music Engineering, University of Miami, Coral Gables, Florida, United States of America; 4 Department of Electrical & Computer Engineering, University of Miami, Coral Gables, Florida, United States of America; 5 Department of Psychology, Florida International University, Miami, Florida, United States of America; 6 Department of Educational and Psychological Studies, University of Miami, Coral Gables, Florida, United States of America; University of Manchester, UNITED KINGDOM

## Abstract

Although looking time is used to assess infant perceptual and cognitive processing, little is known about the temporal structure of infant looking. To shed light on this temporal structure, 127 three-month-olds were assessed in an infant-controlled habituation procedure and presented with a pre-recorded display of a woman addressing the infant using infant-directed speech. Previous individual look durations positively predicted subsequent look durations over a six look window, suggesting a temporal dependency between successive infant looks. The previous look duration continued to predict the subsequent look duration after accounting for habituation-linked declines in look duration, and when looks were separated by an inter-trial interval in which no stimulus was displayed. Individual differences in temporal dependency, the strength of *associations* between consecutive look durations, are distinct from individual differences in mean infant look duration. Nevertheless, infants with stronger temporal dependency had briefer mean look durations, a potential index of stimulus processing. Temporal dependency was evident not only between individual infant looks but between the durations of successive habituation trials (total looking within a trial). Finally, temporal dependency was evident in associations between the last look at the habituation stimulus and the first look at a novel test stimulus. Thus temporal dependency was evident across multiple timescales (individual looks and trials comprised of multiple individual looks) and persisted across conditions including brief periods of no stimulus presentation and changes from a familiar to novel stimulus. Associations between consecutive look durations over multiple timescales and stimuli suggest a temporal structure of infant attention that has been largely ignored in previous work on infant looking.

## Introduction

**Fig 1:**
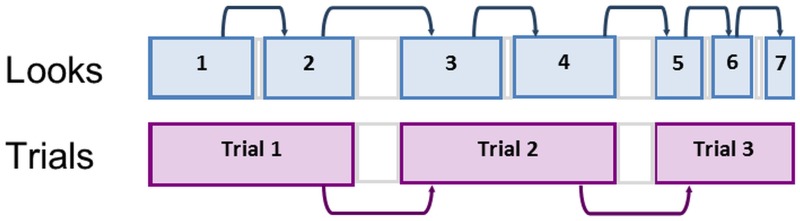
An illustration of the temporal dependency hypothesis. Individual looks occur within trials of the habituation protocol. Individual look durations positively predict subsequent look durations. Previous trial durations predict subsequent trial durations.

Much research on early cognition, perception, and learning relies on assessing overall looking times for groups of infants. Although overall looking times involve a sequence of individual visual looks toward a target separated by brief looks away, little attention has been paid to the succession of individual look durations. In studies employing habituation, for example, results are typically aggregated across individual looks within a trial and reported in terms of overall looking times across trials (see [1–4]). Here we investigate the temporal structuring of individual infant fixations or looks at a stimulus. We test the temporal dependency hypothesis: the duration of an infant’s look will positively predict the duration of the infant’s next look over a variety of timescales (see Fig 1). This hypothesis is motivated by a dynamic systems perspective which focuses on the interrelationship of factors affecting sequences of individual actions [5,6]. Findings of temporal dependency would suggest infant organization of their own visual exploration and shed new light on behavioral dynamics during widely-used assessments of infant cognition.

### Temporal dependency

Temporal dependency in the durations of consecutive adult actions has been documented for reaction times, judgments of time intervals, and other cognitive operations, suggesting robust patterning of the durations of consecutive human behaviors [7–9]. Yet there is no parallel literature on temporal dependency between infant looks in experimental contexts. A relevant study of face-to-face interaction indicated that the duration of infant looks to the mother’s face were predicted by the durations of the two previous looks at her face [10]. This temporal dependency finding, however, might have been due to the effects of mother behavior on infant looking. By contrast, the current investigation examined infant looks at a prerecorded social stimulus, precluding the possibility of that the social partner was contributing to temporal dependency in infant behavior.

A rich literature on infant looking in experimental contexts informs the temporal dependency hypothesis. Research with one-month-olds looking at physical objects [11] and 6- to 24-month-olds watching children’s movies [12] suggests *within-look* inertia that prolongs a gaze at a stimulus once a gaze has been initiated. Time-series analyses, which use a specific interval as a unit of analysis, also provide evidence for the temporal structuring of looking. Fisher-Thompson [13] found that successive intervals (ranging from .25 to 10.0 seconds) of gazing in a two-screen task to one side or another show autocorrelation, which contributes to short-term side-bias. However, these investigations did not examine associations between the durations of successive infant-initiated looks.

Previous investigators have modeled habituation processes—a decline in looking during repeated exposure to a stimulus [14,15]—to predict overall looking in trials and other epochs of continuous stimulus presentation. Although theorists have proposed models of habituation in which dynamic processes yield continuity in looking time over successive trials, these models do not speak to individual looks [16–18]. Perone and Spencer [19,20] propose models of reciprocal excitatory and inhibitory associations which produce reliable simulations of the number of looks and look durations within trials in a habituation task. However, model-fit was evaluated with respect to trial-aggregated parameters, leaving open the question of temporal dependency between individual infant looks within trials.

The current investigation emphasizes individual infant-initiated looks as a unit of analysis based on the premise that infant-initiated actions are key to understanding attention and related processes [21]. An individual look was defined as a period of visual fixation of the stimulus ending with a look away from the stimulus as determined on-line by a human observer. Individual looks are a finer measure of duration than trial duration (the total duration of individual looks within a trial, e.g., [22,23]); they are not as fine a measure as discrete fixation durations assessed with eye trackers [24,25]. To provide continuity with previous studies using visual habituation procedures, and to motivate prediction of looking in test trials, we tested for temporal dependency not only between individual looks but between trials of looking. See Fig 1 for a representation of temporal dependency between individual looks and between trials.

### Temporal dependency and habituation

A central question was whether previous look durations predicted next look durations even after accounting for habituation-related declines in look durations. Visual habituation involves a reduction in looking over repeated presentations of a stimulus and is thought to index stimulus encoding (for reviews, see [4,15,26]). In the current study, the temporal dependency hypothesis was tested during repeated presentations of a pre-recorded social display within an infant-controlled habituation procedure which is typically used to account for individual differences in habituation rate [14,27,28]. In analyses, habituation was modeled as an infant-specific (Gilmore & Thomas, 2002) decline in the durations of individual looks within and across trials of the protocol. *Temporal dependency and habituation were hypothesized to uniquely contribute to the prediction of individual look durations*.

### Individual differences in mean look duration

We used multilevel modeling to assess the extent to which temporal dependency and habituation affected the durations of individual looks within individual infants. Individual differences in mean look durations are instantiated in multilevel models by random variance (between infants) in the intercept term. Consequently between-infant differences in mean look durations did not influence the estimation of temporal dependency, allowing us to examine empirical associations between these variables. Substantively, shorter mean infant look duration has been associated with more comprehensive stimulus processing as indexed by greater discrimination of global and featural information [29] and reduced time to demonstrate a novelty preference [30]. Consequently, we investigated whether individual differences in temporal dependency, the degree to which infants constrain their own look durations, was associated with mean look duration an index of stimulus processing.

### Temporal dependency between stimuli and trials

Temporal dependency may reflect infants’ organization of their own visual exploration or features of the stimulus. To assess the potential impact of stimulus characteristics on temporal dependency, we compared presentation of a moving face with naturally occurring voice (audiovisual) with presentation of the face alone (silent video). To test the robustness of temporal dependency, we also examined associations between successive infant looks that were separated by an inter-trial interval. We first focused analysis on pairs of looks that spanned the three second inter-trial interval in the habituation phase of the protocol to ascertain the impact of inter-trial intervals on temporal dependency. Previous modeling suggests an association between the last look of one trial and the first look of the next trial is unlikely because the inter-trial interval is likely to disrupt ongoing attention processes [16]. We also created a more drastic test of temporal dependency by asking whether the duration of the last look at the habituation stimulus predicted the first look at the novel test stimulus.

Next we explored whether temporal dependency was present not only between individual looks but between trials of looking (aggregating over individual looks), a standard unit of analysis in studies using habituation procedures [2,15,22,23]. A specific goal was to assess whether the last trial of looking to the habituation stimulus predicted looking to the novel test stimulus. That is, we asked whether temporal dependency was operative at the level of trials of looking despite a change in the stimulus being presented.

Finally, we asked whether individual differences between infants in the strength of temporal dependency—both between consecutive looks and consecutive trials—were associated with mean look duration and the slope of habituation related declines in looking, which index infant processing of the habituation stimulus. We also measured the association between temporal dependency and visual recovery. Visual recovery to a new test stimulus (presented after habituation) is designed to index infant discrimination of the stimuli’s novel features [3,29].

### Overview

The overall study goal was exploration of the temporal dependency hypothesis positing a link between the durations of consecutive looks. We began by describing the limits of temporal dependency, asking whether a given look, the *n*th in a series of looks, was predicted by the duration of the previous look, the look before that, and so on. We next asked whether successive individual look durations exhibited temporal dependency after accounting for habituation effects. Having accounted for unique effects of temporal dependency and habituation, we tested whether infants with stronger temporal dependency, tighter associations between consecutive looks, would exhibit shorter mean look durations, a potential index of stimulus processing. Next we ascertained the robustness of temporal dependency by examining associations between consecutive looks that spanned the inter-trial intervals of the habituation protocol, asking whether the duration of the last look at the habituation stimulus predicted the duration of the first look at a novel test stimulus, and determining whether temporal dependency was present between the durations of trials of looking, a standard unit of analysis in habituation studies. Finally, we asked whether individual differences in temporal dependency were associated with differences in habituation slope or visual recovery.

## Method

### Participants

Participants were 127 three-month-olds (*M* = 99.83 days, *SD* = 13.80; 57 females). All infants were healthy and born full-term, weighing at least five pounds, with APGAR scores of at least 9. Eighty-eight infants (69%) received a visual display with a synchronized soundtrack of a woman speaking and 39 infants (31%) received visual displays of a woman speaking with no soundtrack. Demographic information is presented separately for infants who received the audiovisual and visual only presentations (see Table 1).

**Table 1:** Sample demographics.

Presentation Type	N	Mean Age in Days (SD)	Male	Non-Hispanic Caucasian	Hispanic	African-American/Asian/Other
Audiovisual	88	103.34 (14.96)	41	4	81	3
Visual Only	39	91.90 (5.11)	17	5	33	1
Combined	127	99.83 (13.80)	58	9	114	4

These 127 infants were part of a larger study in which mean performance for a variety of variables was reported, including total time to habituation, the look-away rate during habituation, the mean duration of the first two habituation trials, mean looking time over test trials, and visual recovery scores [2,31]. The durations of infants’ individual looks and the durations of total looking times within individual trials—the focus of the current study—have not been published. Study protocols were approved by the Florida International University Institutional Review Board. Before participating, informed consent was obtained in writing from the infants' parent or legal guardian.

### Stimuli

#### Habituation

Dynamic color video displays of infant-directed speech served as the stimulus events [2,32–34]. Three actress’ faces and shoulders were videotaped as they recited “Mary Had a Little Lamb” (18 s) and “Jack and Jill” (12 s). The alternating rhymes were looped into a single video which contained either the original face-voice combination or a face synchronized with another actress’ voice. Each infant was presented with a single video (one actress’ face). The visual component of the videos was identical across the audiovisual (moving face producing synchronous infant-directed speech) and silent visual (moving face speaking silently) conditions.

#### Test

Single screen test stimuli—combinations of the previously-described social videos—were presented to 83 infants (*M* = 3.50 months, *SD* = 0.48) in two test trials. During these test trials, 35 infants were presented with a novel face speaking with a familiar voice to assess face discrimination [2]; 15 infants were presented with a familiar face speaking with a novel voice to assess voice discrimination [33]; and 33 infants were presented with a novel face-voice combination to assess face-voice discrimination [34]. Visual recovery was calculated by subtracting the mean duration of the final two habituation trials from the mean duration of the two test trials.

### Apparatus

Videos were presented using Panasonic video players (DS545 and AG7750) and displayed on a 19 inch color monitor (Sony KV-20520). Infants sat approximately 55 cm from the monitor in a standard infant seat. Black curtains surrounded the television monitor to obscure extraneous stimuli and two 1.5-cm apertures allowed trained observers to view the infants’ visual fixations. All soundtracks were presented from a centrally-located speaker. Observers used button presses to record the length of the infants’ visual fixations and these data were collected on digital media. The primary observer’s observations controlled the stimulus presentations and the secondary observer’s observations were used in calculating inter-observer reliability.

### Procedure

A single-screen, infant-controlled habituation procedure was utilized as a context in which to test for the presence of temporal dependency and habituation effects on individual looks. Trials began when the infant fixated the video image and lasted until the infant looked away for 1.5 seconds or until the maximum trial length of 60 seconds had elapsed. Less than a tenth of trials reached this maximum length (*M* = .09, *SD* = .14). There was a 3 second inter-stimulus interval between trials. Attention–grabbing stimuli were not used between trials. All infants in the dataset reached habituation such that the mean of their overall looking (the sum of looking time at the stimulus) in two consecutive trials declined to 50% or less of their overall looking in the first two trials of the procedure. After reaching habituation, infants received an additional two habituation trials of the original habituation stimulus to rule out error-related recovery when novel test stimuli were displayed [35].

### Individual looks

Individual looks began when infants visually fixated the video image. Looks ended when an infant shifted their eyes away from the video image for at least .2 seconds. Consequently, the durations of individual looks were periods of visual fixation of the video image that might contain instances in which the infant looked away from the video image for less than .2 seconds. A look away of 1.5 seconds terminated a trial. A primary observer’s coding determined all looking events and durations. A second observer was available for 20% of the infants. The mean intra-class correlation between the primary and secondary observers’ measurements of individual look durations was .83 (*SD* = .14). This indicates relatively high consistency in the coding of infants’ individual look durations. In addition, prior to coding, all observers were trained to reliability (coding 10 consecutive participants with a correlation of .90 or greater with a previously trained observer on total looking time per trial).

### Data analysis

Multilevel modeling was used to predict the durations of successive individual looks (level 1) within the sample’s 127 infants (level 2). Multilevel models are conceptually similar to conducting a multiple regression for each infant in which each consecutive look is predicted by its previous look. The results of the individual predictions for each infant are amalgamated to ascertain the significance of previous look duration in the sample. The significance of multiple predictors may be assessed in the same model. Multilevel modeling was conducted using the Hierarchical Linear and Nonlinear Modeling (Version 6.06) software [36]. All predictor variables—main effects and interactions—were centered around their within-infant means [37]. This ensured that between-infant effects (e.g., the mean look durations of different infants) did not influence the modeling of individual look durations within infants.

Multilevel parameters include fixed effects (an average effect over infants) and random effects (individual infant variance around the fixed effect). Although modeling focused on fixed effects, random effects were included for all effects assessed at level 1 (within infants). All models employed full-information maximum likelihood estimation using an unstructured (full) variance/covariance matrix [38]. Deviance statistics index the fit of the modeled data to the fit of the observed data provided by a saturated model. To select among nested models, chi-square difference tests were used with the additional criterion that *t-*tests of fixed effect parameters be statistically significant. This allowed selection of parsimonious models whose parameters could be substantively interpreted. Percent of Variance Accounted For (PVAF), a pseudo measure of effect size, was calculated as variance accounted for in a nested model including an effect of interest in comparison to the model without that effect.

## Results

After describing the number and duration of individual looks, we examined the extent of temporal dependency, the number of previous or lagged looks associated with the current look. Then, the unique effects of temporal dependency and habituation were assessed. This laid the groundwork for a group level analysis of the association of temporal dependency strength and mean look length between infants. Temporal dependency effects—associations between consecutive individual looks—were examined over the inter-stimulus interval between trials, and in the transition from looks at the habituation stimulus to looks to the novel test stimulus. Temporal dependency was then tested between trials of looking and used to predict the duration of the first trial of looking to the novel test stimulus. Finally, we examined associations between individual differences in temporal dependency and mean length of looking, strength of habituation, and visual recovery to a novel stimulus.

### Description of looks and trials

The number and duration of individual looks and trials in the habituation protocol is described in Table 2. The mean overall duration of individual infant looks was 9.3 seconds and there were a mean of 27.8 individual looks during the protocol. There was a mean of 2.6 looks per trial, and trials had a mean duration of 24.4 seconds. The mean number of trials—habituation trials plus the two post-habituation trials—was 10.6. The audiovisual and the visual only conditions did not differ on any of these metrics (*p*s >.5) and analyses included looks from both conditions (see Table 2).

**Table 2:** Habituation trials and individual looks.

Presentation Type	N	Mean Look Duration (SD)	Mean Number of Looks (SD)	Mean Number of Looks per Trial (SD)	Mean Trial Duration (SD)	Mean Number of Trials (SD)
Audiovisual	88	9.46 (12.34)	27.72 (14.31)	2.62 (2.34)	24.75 (21.55)	10.59 (3.07)
Visual Only	39	8.78 (11.30)	28.00 (12.70)	2.67 (1.99)	23.41 (20.70)	10.56 (3.08)
Combined	127	9.25 (12.03)	27.80 (13.79)	2.63 (2.24)	24.36 (21.29)	10.58 (3.06)

Durations are in seconds. The mean number of trials includes the habituation trials and two post-habituation trials.

The audiovisual and the visual only conditions did not in the number of trials per infant, *t*(125) = .05, *p* = .96, the number of individual infant looks to the stimulus, *t*(125) = -0.17, *p* = .87, or their mean duration, *t*(125) = 0.58, *p* = .56.

Look durations followed a lognormal distribution and all durations and counts were log-transformed (*log10*[*x* + 1]) prior to analyses [10,12]. This reduced the skew of individual look durations (2.43 to .38) and look counts (1.20 to .04), as well as trial durations (.68 to -.28) and trial counts (1.37 to .91). The log-transformation provided a common metric for all look measures.

### The extent of temporal dependency?

We first tested the temporal dependency hypothesis by assessing the impact of previous look durations on current look duration in a series of models. We began with an empty model containing an intercept, the fixed effect of mean look duration, and a random effect specifying infant-specific differences in that mean. The empty model controlled for individual differences in infant mean look durations. It provided a basis of comparison for models containing temporal dependency terms.

**Fig 2:**
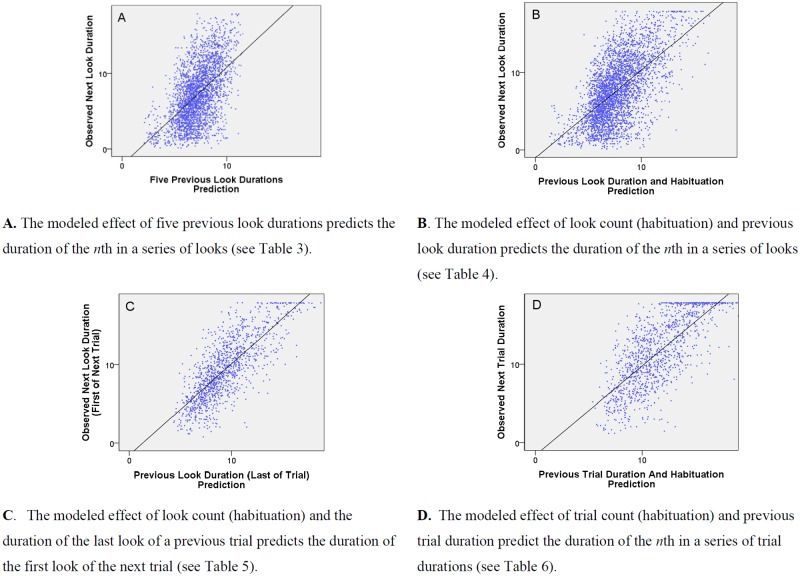
Temporal dependency. Individual look durations are predicted by previous individual look durations (A–C) and trial durations are predicted by the previous trial duration (D). Durations are displayed in seconds on a log 10 scale. **A.** The modeled effect of five previous look durations predicts the duration of the *n*th in a series of looks (see Table 3). **B.** The modeled effect of look count (habituation) and previous look duration predicts the duration of the *n*th in a series of looks (see Table 4). **C.** The modeled effect of look count (habituation) and the duration of the last look of a previous trial predicts the duration of the first look of the next trial (see Table 5). **D.** The modeled effect of trial count (habituation) and previous trial duration predict the duration of the *n*th in a series of trial durations (see Table 6).

The durations of successive previous looks (e.g., one look back, one and two looks back, etc.) were used to predict the current look duration in a series of iteratively fit models (see S1 Table). This yielded a model with five previous looks with significant fixed effects (see Table 3 and Fig 2A). The durations of each of an infant’s five previous looks were uniquely associated with the duration of the current look, constituting a system of temporally linked looks. Associations were positive such that longer previous looks predicted longer current looks.

**Table 3:** Model predicting look duration with five previous looks (lags).

	Fixed Effects					Random Effects				
Model Parameters	*Β*	*SE*	*T*	*df*	*p*	*Variance*	*SD*	*χ*^*2*^	*df*	*P*
Intercept	0.75	0.01	53.78	126	< .001	0.02	0.14	588.31	122	< .001
Lag 1 Duration	0.10	0.02	4.64	126	< .001	0.02	0.13	150.55	122	.040
Lag 2 Duration	0.05	0.02	2.69	126	.009	0.01	0.07	121.66	122	> .50
Lag 3 Duration	0.05	0.02	2.24	126	.027	0.01	0.11	138.93	122	.140
Lag 4 Duration	0.05	0.02	2.24	126	.027	0.01	0.12	130.11	122	.291
Lag 5 Duration	0.09	0.02	5.20	126	< .001	0.01	0.05	101.32	122	> .50

The model describes the unique effects of the durations of previous five looks on the duration of the *nth* in a series of looks. Lag 1 refers to the immediately previous look, Lag 2 to the look previous to that, and so on until Lag 5. The model’s equation and unstructured (full) covariance matrix are reported in S2 Table.

### Temporal dependency and habituation

In addition to temporal dependency, individual look durations are affected by habituation which depresses the durations of successive looks. Do temporal dependency effects on individual looks remain significant after accounting for the impact of habituation? To address this question, habituation was operationalized as the log of the count of individual looks. (See S1 Table for a comparison of alternate habituation measures). Within predictive models, the habituation term indexes a logarithmic decline in look duration over successive looks. The variance component of the habituation term allowed for individual differences between infants in the decline in look durations.

We asked whether the temporal dependency effect—the influence of the previous look duration on the current look duration—remained significant while controlling for habituation (see Table 4). Adding the effect of the first previous look duration to a model containing the habituation term yielded a significant improvement in model fit, χ^2^(4) = 100.66, *p* <.001. Although adding a term for the second previous look (lag 2) also yielded an improvement in model fit, χ^2^(5) = 119.26, *p* <.001, the fixed effect of the second previous look was not significant, β = -.01, *t*(126) = -0.37, *p* = .714. Consequently, a model containing the independent effects of the habituation term and a single temporal dependency term (the previous look) was maintained. Model parameters are described in Table 4 and their effects are graphed in Fig 2B. In this final model, two processes uniquely affected look durations. Look durations tended to decline over successive individual looks (habituation), and looks of a given duration tended to predict looks of a similar duration (temporal dependency).

**Table 4:** Final model predicting individual look durations.

	Fixed Effects					Random Effects				
**Model Parameters**	***Β***	***SE***	***t***	***df***	***p***	***Variance***	***SD***	***χ***^***2***^	***df***	***p***
Intercept	0.83	0.02	53.89	126	< .001	0.02	0.16	866.46	126	< .001
Log10 Look Count	-0.51	0.03	-14.94	126	< .001	0.07	0.27	314.48	126	< .001
Lag 1 Duration	0.05	0.02	2.50	126	.014	0.01	0.08	150.48	126	.068
**Model Fit Statistics**	**Deviance**	**Number of Parameters**		**Level 1 Observations (Individual Looks)**		***χ***^***2***^	***p***	***AIC***		***BIC***
	2546.53	10		3409		100.66	< .001	2566.53		2581.86

Individual look duration is uniquely predicted by log10 look count (habituation) and lag 1 duration (temporal dependency). The *χ*^*2*^ describes a comparison to a model with no temporal dependency (Lag 1) effects (see text). The inclusion of Log10 Look Count as a predictor accounted for 27.0% more variance than a model with the intercept alone. The inclusion of Lag 1 Duration as a predictor accounted for 0.6% more variance than a model with Log10 Look Count. For the model’s equation and unstructured (full) covariance matrix see S1 Text.

### Modeling the audiovisual and visual only condition comparison

Is temporal dependency affected by characteristics of the stimulus presented? To address this question, we ascertained whether the association between previous and current look durations differed as a function of type of presentation (audiovisual, *n* = 88, or silent visual, *n* = 39). Adding type of presentation and its interactions with the temporal dependency and habituation terms did not yield an improvement in model fit over a model containing only the temporal dependency and habituation terms, *χ²*(3) = 1.17, *p*>.50. Interpretation of the nonsignificant interaction should be tempered by awareness of a potential lack of power caused by unequal group sizes. Nevertheless, the lack of interaction suggests that temporal dependency was not contingent on stimulus characteristics, and type of presentation was not included in subsequent analyses.

We next asked whether temporal dependency was associated with infant gender or age. Adding gender and its interactions with the temporal dependency and habituation terms to the final model (see Table 4) did not yield an improvement in model fit, χ^2^(3) = 4.89, *p* = .18. Likewise, adding age in days and its interactions with temporal dependency and habituation terms did not yield an improvement in model fit, χ^2^(3) = 1.11, *p*>.50.

### Temporal dependency of individual looks between habituation trials

To better understand the robustness of the temporal dependency effect, we examined associations between the previous look and next look across the inter-stimulus interval between trials. That is, we analyzed the association between the last look of one trial and the first look of the next trial (see Table 5). We first constructed an empty model in which the durations of the first look of trials were predicted by the group mean (the intercept) of the duration of these first looks and individual variance around that mean. There were successive improvements in model fit after adding both the habituation term (log-transformed look count), χ²(3) = 568.65, *p* <.001, and the temporal dependency term (previous look duration), χ²(4) = 146.26, *p* <.001. The durations of the first look in a trial were significantly predicted by the duration of the last look of the previous trial while accounting for significant habituation effects (see Table 5). Previous look durations positively predicted subsequent look durations even after an inter-stimulus interval of 3 seconds (see Fig 2C). This suggests the robustness of the temporal dependency effect across a brief period in which no target is present.

**Table 5:** Final model predicting the first individual look of a habituation trial.

	Fixed Effects					Random Effects				
**Model Parameters**	***Β***	***SE***	***t***	***df***	***p***	***Variance***	***SD***	***χ***^***2***^	***df***	***p***
Intercept	0.98	0.02	58.14	126	< .001	0.03	0.16	450.26	126	< .001
Log10 Look Count	-0.62	0.04	-14.53	126	< .001	0.07	0.27	178.42	126	.002
Lag 1 Duration	0.07	0.03	2.55	126	.012	0.03	0.17	162.01	126	.017
**Model Fit Statistics**	**Deviance**	**Number of Parameters**			**Level 1 Observations (Individual Looks)**		***χ***^***2***^	***p***	***AIC***	***BIC***
	595.16	10			1215		146.26	< .001	615.16	626.01

The duration of the first individual look of a trial is uniquely predicted by log10 look count (habituation) and lag 1 duration (temporal dependency). The model uses the last look of one trial to predict the first look of the next trial in the habituation protocol. The *χ*^*2*^ describes a comparison to a model with no temporal dependency (Lag 1) effects (see text). The inclusion of Log10 Look Count as a predictor accounted for 40.8% more variance than a model with the intercept alone. The inclusion of Lag 1 Duration as a predictor accounted for 10.7% more variance than a model with Log10 Look Count. For the model equation and unstructured (full) covariance matrix see S1 Text.

### Temporal dependency in individual looks over habituation and test stimuli

Having established predictive associations over the inter-stimulus interval, we asked whether temporal dependency was also evident across changes in the stimulus display. To wit, was there an association between the last individual look at the habituation stimulus and the first look at the novel test stimulus? Overall, last looks at the habituation stimulus were briefer (*M* = 5.00 seconds, *SD* = 5.02) than first looks at the test stimulus (*M* = 10.46, *SD* = 11.15), *t*(82) = 4.72, *p* <.001. There was nevertheless a significant correlation between the duration of the last look at the habituation stimulus and the duration of the first look at the test stimulus, *r*(81) = .30, *p* = .005. Moreover, in a regression model controlling for whether infants were short or long lookers (b = .05, *p* = .60), the duration of the last look at the habituation stimulus continued to be associated with the first look at the novel test stimulus, b = .34, *p* = .003. That is, previous individual looks at one stimulus predicted subsequent looks at the new stimulus.

### Temporal dependency between the durations of habituation trials

While temporal dependency was apparent between individual infant looks, the trial is the standard unit of analysis for habituation protocols. To test for temporal dependency between the durations of successive habituation trials, we constructed an empty model in which trial duration was predicted by the group mean (the intercept) of trials and individual variance around that mean. There were improvements in model fit after sequentially adding both the habituation term (log-transformed trial number), χ²(3) = 566.73, *p* <.001, and the temporal dependency term (previous trial duration), χ²(4) = 116.73, *p* <.001. Trial duration was uniquely predicted by both habituation and temporal dependency terms (see Table 6 and Fig 2D). In one fifth of trials (20.2%), the previous and current trial each contained only a single look. Thus the current analysis is not independent the analysis of individual looks. The results nevertheless suggest that temporal dependency is operative both at the level of individual looks and between habituation trials (which typically involve multiple looks).

**Table 6:** Final Model Predicting Trial Durations.

	Fixed Effects					Random Effects				
**Model Parameters**	***Β***	***SE***	***T***	***df***	***p***	***Variance***	***SD***	***χ***^***2***^	***df***	***p***
Intercept	1.17	0.02	60.26	126	< .001	0.04	0.19	574.63	126	< .001
Log10 Trial Count	-0.81	0.06	-14.11	126	< .001	0.07	0.27	136.43	126	.248
Lag 1 Duration	0.18	0.03	5.39	126	< .001	0.02	0.14	116.07	126	> .50
**Model Fit Statistics**	**Deviance**	**Number of Parameters**		**Level 1 Observations (Trials)**			***χ***^***2***^	***p***	***AIC***	***BIC***
	903.47	10		1217			116.73	< .001	923.47	934.32

Trial looking duration is uniquely predicted by log10 trial count (temporal dependency) and lag 1, the previous trial duration (temporal dependency). The *χ*^*2*^ describes a comparison to a model with no temporal dependency (Lag 1) effects (see text). The inclusion of Log10 Trial Count as a predictor accounted for 38.6% more variance than a model with the intercept alone. The inclusion of Lag 1 Duration as a predictor accounted for 5.6% more variance than a model with Log10 Trial Count. For the model’s equation and unstructured (full) covariance matrix see S1 Text.

### Temporal dependency in trials between habituation and test stimuli

To further assess the impact of temporal dependencies over stimuli we focused on associations between the duration of the last trial of looking to the habituation stimulus and the first trial of looking to the novel stimulus. As expected, the duration of the last habituation trial was briefer (*M* = 9.73 seconds, *SD* = 6.23) than the first trial of looking at the novel test stimulus (*M* = 15.19, *SD* = 12.12), *t*(82) = -3.68, *p* <.001. Nevertheless the duration of the last trial of looking to the habituation stimulus was positively associated with the duration of looking in the first test trial, *r*(81) = .23, *p* = .036. Moreover, in a regression model controlling for whether infants were short or long lookers (b = -.03, *p* = .76), the duration of the last habituation trial continued to be associated with the duration of the first trial of looking at the novel test stimulus, b = .29, *p* = .006. That is, consecutive trial durations were associated even when the stimuli to which the infant was attending had changed.

### Individual differences in the strength of temporal dependency

**Fig 3:**
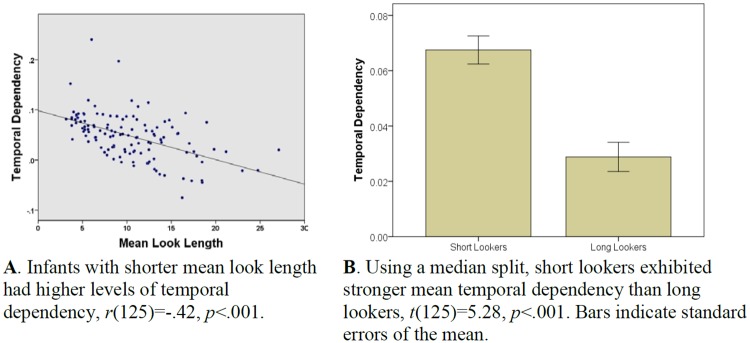
Individual infants’ temporal dependency parameters were derived from the final multilevel model (Table 4). **A.** Infants with shorter mean look length had higher levels of temporal dependency, *r*(125) = -.42, *p* <.001. **B.** Using a median split, short lookers exhibited stronger temporal dependency than long lookers, *t*(125) = 5.28, *p* <.001. Bars indicate standard errors of the mean.

We next asked whether individual differences in temporal dependency were associated with individual differences in looking time and novelty preference. To examine the association between temporal dependency and looking time, we derived individual infants’ temporal dependency parameters from the final multilevel model and calculated mean look length over the habituation protocol. Among individual infants, mean look length was negatively associated with the strength of temporal dependency, *r*(125) = -.42, *p* <.001 (see Fig 3a). When a median split was applied, short lookers exhibited higher mean temporal dependency values than long lookers (*M* = .07, *SD* = .04 vs. *M* = .03, *SD* = .04), *t*(125) = 5.28, *p* <.001 (see Fig 3b). On average, then, infants with shorter looks had high levels of temporal dependency.

Using temporal dependency values from the appropriate final models, and novelty preference to the several test stimuli to which the 83 infants with relevant data had been exposed [2,33,34], we calculated associations between these variables. Neither individual differences in the strength of temporal dependency between successive individual looks: *r*(81) = -.07, *p* = .556 or between the durations of successive trials, *r*(81) = -.04, *p* = .751, was associated with novelty preference (discrimination). Individual differences in the strength of temporal dependency at the level of looks were not associated with individual differences in habituation at the level of looks, *r*(125) = .08, *p* = .387. However, infants with stronger temporal dependency between consecutive trials exhibited faster habituation, more negative declines in trial duration over successive trials, *r*(125) = -.54, *p* <.001.

## Discussion

We found strong evidence for a temporal dependency effect structuring infant looking behavior. There was a robust association between the durations of previous and subsequent looks in a large sample of young infants assessed in a widely-used infant-controlled habituation procedure. A single lag dependency was found in which previous looks predicted subsequent looks 1) after accounting for habituation, 2) over an inter-stimulus interval, and 3) in the transition from a familiar to a novel stimulus. These results suggest that past infant action affects subsequent action. Such findings are consonant with a dynamic systems emphasis on the influence of past action on subsequent action, but they have not been previously documented in the extensive literature on infant looking. The relevance of the results for understanding infant looking behavior and studying attention are discussed below.

### Infant looking and temporal dependency

To better understand perceptual and cognitive development—and their interface with real-time learning—researchers have examined peak look durations [39], mean look length [40], forced-preference judgments [41], first look direction [42], the temporal course of individual looks [11], the lognormal distribution of look durations [12], the frequency of look shifts in two-screen displays [20], and the frequency of look shifts to specific regions of a stimulus [43,44]. More generally, analysis of the moment-to-moment structure of infant behavior has led to unique insights into infant learning in a variety of domains [45,46]. There has, however, been little attention to successive, individual look durations (observer-coded periods of infant gazing at the stimulus separated by brief gazes away from the stimulus).

We examined temporal or serial dependencies in infant looking to a dynamic social stimulus during a habituation protocol, which are frequently used to assess infant perception, learning, and cognitive processing. Results extend findings that infants’ previous looks predict successive looks at mother’s face during a face-to-face interaction (Messinger et al., 2012). Findings of parallel processes between social interaction and habituation protocols are relatively rare. Convergent findings of temporal dependency across both contexts reveal the robustness of the phenomenon.

### The extent of temporal dependency

An impressive number of preceding looks, five, significantly predicted unique variance in the duration of the current look. Each look had an independent, additive effect on the current look duration. That is, any given look was temporally bound to previous looks (that it was predicted by) and to subsequent looks (that it predicted). These temporal dependency effects tend to produce periods of longer looks and periods of briefer looks. The findings are reminiscent of predictive associations between the durations of lagged reaction times and mental rotation tasks in adults [47–49]. In both cases, the characteristics of behaviors used to assess perceptual and cognitive processing propagate through time.

### Temporal dependency and stimulus characteristics

In the current study infants engaged with a prerecorded social stimulus. It is nevertheless possible that the stimulus contained epochs that were more or less interesting, which might contribute to temporal dependency effects. However, the probability that stimulus properties are responsible for the current temporal dependency effects is low for several reasons. First, temporal dependency was apparent between looking at the habituation stimulus and looking at the novel test stimulus. Second, temporal dependency effects in response to audiovisual and visual only stimuli were not distinguishable. Finally, temporal dependency effects were apparent between trials (over the 3 second inter-stimulus interval when the video stimulus was not visible or audible). These findings suggest that temporal dependency reflects infant structuring of their visual engagement with the environment (see [12]). Nevertheless, additional research with a range of stimuli—including nonsocial displays and static displays—at a range of ages is necessary to more fully address this issue.

### Temporal dependency and habituation

Temporal dependency provides insight into looking time dynamics in habituation protocols [14–19,50]. Habituation involves a decrement in behavioral response over repeated exposure to a stimulus. In the current analyses, temporal dependency effects persisted after accounting for habituation. Gilmore and Thomas (2002) make common assumptions explicit in a formal model of habituation. In their model, all deviations from infant-specific habituation curves are categorized as error. A portion of the error in previous habituation model, however, becomes explained variance in the current model. After accounting for habituation, variance in look durations was further reduced by accounting for previous look durations. Previous individual look duration accounted for .6% of additional variance in all look durations and 10.7% of the variance in the duration of the first look of a trial. The previous trial duration accounted for 5.6% of additional variance in predicting subsequent trial durations.

Habituation and temporal dependency terms quantify distinct processes. Habituation describes a long range relationship affecting the entire series of looks (or trials) while temporal dependency describes a local association between successive look durations. Habituation produced an overall decrease in look duration for each successive look. Within that context, temporal dependency constrained pairs of successive looks to be similar in length such that relatively long or short looks were followed by looks of a similar duration. On a broader level, the current results suggest that individual looks are a promising, data-rich unit of analysis that can be used to complement analyses at the level of trials in investigations of infant perception and cognition.

### Temporal dependency of individual looks between habituation trials

In a demonstration of the robustness of temporal dependency, the duration of the final look in a trial predicted the duration of the first look in the next trial. That is, the predictive association between the durations of consecutive looks was present over the inter-trial interval, a period predicted to introduce discontinuities in looking time [16–18]. Although last looks in a trial were approximately half as long as first looks to the re-presented stimulus, the three second inter-stimulus interval did not reset attentional dynamics. Instead, temporal dependency bridged the inter-stimulus interval, suggesting its robustness. The robustness of temporal dependency was also illustrated in the transition to looks at the test stimulus.

### Temporal dependency between habituation and test stimuli

In visual habituation procedures, infant perceptual and cognitive capacities are evident when declines in infant looking to a repeatedly presented stimulus are reversed with the presentation of a novel test stimulus [51,52]. Despite such a rise, the duration of the last look to the habituation stimulus was positively associated with the duration of the next look to a different stimulus. That is, temporal dependency linked the last look of the habituation trial (the familiarized stimulus) with the first look to the novel test stimulus.

Temporal dependency between stimuli was evident not only at the level of individual looks but over infant controlled habituation trials comprised of multiple looks. The duration of the last trials of looking at the habituation stimulus was associated with the duration of the first trial of looking at the test stimulus. In sum, temporal dependency, at both the level of individual looks and of trials, was evident despite a change in the stimulus presented to the infant.

### Individual differences in temporal dependency

Individual differences in temporal dependency reflect the degree to which infants constrain their own looking durations, the degree to which one look predicts the next. We found an inverse association between temporal dependency and look length. Likewise, a median split indicated that short lookers exhibited higher temporal dependency levels than long lookers. These findings may suggest that infants who exhibit higher temporal dependency levels engage in more efficient stimulus processing than other infants. Neither temporal dependency between successive individual looks or between the durations of successive trials was associated with novelty preference (discrimination). However, different infants encountered different test stimuli such that the difficulty of the discrimination task varied among infants [2,33,34]. Research investigating the association between temporal dependency and independent, consistent assessments of processing is necessary to better understand the relationship of temporal dependency to perceptual and cognitive functioning.

At the level of looks, individual differences in temporal dependency and habitation were not associated. At the individual look level, then, temporal dependency is not dependent on the strength of habituation-related declines. Temporal dependency describes a micro-structure, the tethering of two adjacent look durations; habituation describes a macrostructure, the decrease in look durations over an entire stream of looks. At the level of trials, however, infants with stronger temporal dependency exhibited faster habituation. This indicates that infants who habituated more slowly had trial durations with weaker successive links between them. At the trial level, then, weak temporal dependency indexes attenuated structuring of infant looking. The possibility that some infants exhibit both less short-range and long-range temporal structuring than others warrants further research and suggests the importance of individual differences in studies of infant perception and cognition.

### Sequences of behavior

The autocorrelation component of a time-series is a well-established statistical phenomenon with similarities to temporal dependency. However, there is a crucial distinction between temporal dependency—associations between the durations of successive actions—and autocorrelation. Autocorrelation refers to the association between behavior at a fixed interval in time (e.g., 1 second), *t*, and behavior at a previous interval, *t-1* [13,53,54]. By contrast, temporal dependency involves associations between the durations of consecutive *events* such as looks and trials. In addition, temporal dependency, but not auto-correlation, involves associations between successive looks that are separated by periods in which the infant looks away from the target.

The most general explanation of temporal dependency stems from a dynamic systems focus on action sequences [5,55]. Temporal or serial dependency may arise when an infant (or adult) engages in a series of repeated actions in an environment. The claim is that at closer (e.g., successive) time intervals, an individual is likely to produce more similar actions. For example, just as infant looks of a given duration tend to be followed by looks of a similar duration, repeated manual reaches to a given location tend to be followed by an additional reach to that location (the A-not-B error; [55]). The degree to which such temporal dependencies structure diverse behaviors at different ages is a ripe area for investigation [47].

By definition, temporal dependency involves the impact of previous behavior on current behavior. To the extent that past behavior is a reflection of an individual’s activity, temporal dependency involves some form of auto-regulation [56]. Individual differences in patterns of infant attention and disengagement have been associated with the regulation of social interactions [57], self-regulation [24,58], and executive control [59–61]. Longitudinal research is required to determine whether infant temporal dependency is also associated with individual differences in self-regulation.

### Attention

Recent dynamic field theory models are concerned with processes that affect consecutive look durations. Perone and Spencer, for example, propose reciprocal excitatory and inhibitory associations between perceptual fields and working memory. These modeled associations have produced reliable simulations of the number of looks and look durations over trials in a habituation task [19] and of rate of shifting and individual look durations in a discrimination task [20]. From this perspective, the current temporal dependency results—in which looks of a given duration tend to follow one another—may reflect moment to moment changes in perceptual saliency and short-term-memory processes.

### Future directions

The frequently-utilized habituation procedure imposed constraints on infant looks. Slightly less than one tenth of trials were terminated when a maximum duration of 60 seconds was reached. Free-viewing paradigms could provide a salient future test of the temporal dependency hypothesis and would allow for investigation of longer-range temporal dependencies such as 1/f noise [47]. Temporal dependency effects may also be apparent at finer levels of analysis such as looks to different areas of a stimulus [24,43,44]. Likewise, temporal dependency analyses of infant looking to different objects or activities in naturalistic contexts [62] might reveal those components of a scene the infant perceived to be similar.

In sum, motivated by dynamic systems principles and informed by current models of habituation, we found that individual look durations at a dynamic social stimulus are not randomly determined in the moment. Instead infant actions are organized in a pattern of serial or temporal dependency. Previous look duration predicts next look duration over a succession of looks, suggesting a moving stream of interdependencies in the flow of attention. Uniting literatures on infant and adult actions in temporal context, the results suggest the importance of behavioral history in the investigation of infant learning and development.

## Supporting Information

S1 Dataset Subject-level data. (SAV)

S1 Table Comparison of models containing different numbers of previous looks (lags). (DOCX)

S2 Table 5-lag model covariances. (DOCX)

S3 Table Comparison of alternate habituation models. (DOCX)

S1 Text Model information. (DOCX)
