# Supplementary material for: Temporal Dependency and the Structure of Early Looking
Source: PLoS One. 2017 Jan 11;12(1):e0169458. doi: 10.1371/journal.pone.0169458 (PMC5226676; doi:10.1371/journal.pone.0169458)
Supplement: S1 Table — (DOCX) [file pone.0169458.s002.docx]

| S1 Table. *Comparison of Models Containing Different Numbers of Previous Looks (Lags)* | | | | | | | |
| --- | --- | --- | --- | --- | --- | --- | --- |
| Model | Deviance | Number of Parameters | Level 1 Observations (Individual Looks) | χ^2^ | *p* | AIC | BIC |
| Empty | 3601.21 | 3 | 3536 |  |  | 3607.21 | 3611.86 |
| 1 Lag | 2955.12 | 6 | 3409 | 646.09 | < .001 | 2967.12 | 2976.32 |
| 2 Lag | 2704.30 | 10 | 3282 | 250.83 | < .001 | 2724.30 | 2739.46 |
| 3 Lag | 2452.93 | 15 | 3155 | 251.37 | < .001 | 2482.93 | 2505.41 |
| 4 Lag | 2253.43 | 21 | 3028 | 199.50 | < .001 | 2295.43 | 2326.53 |
| 5 Lag | 2104.03 | 28 | 2901 | 149.39 | < .001 | 2160.03 | 2200.98 |
| 6 Lag | 1948.92 | 36 | 2774 | 155.12 | < .001 | 2020.92 | 2072.87 |

| *Note.* All models contain an intercept, random variance between infants in that intercept, and the fixed and random effects of the specified parameter(s). The empty model has no lags. All models contain the indicated effect and the effects in all previous models. For example, the 2 Lag model contains a Lag 1 and a Lag 2 effect. Lag 1 refers to the immediately previous look, Lag 2 to the look previous to that, and so on. The *χ^2^* reflects comparisons with the previous model, typically the model with one previous lag. The number of looks (level 1 observations) in each successive model declines because each additional lag predictor reduces by one (for each of the 127 infants modeled) the number of looks to be predicted.  The 1 Lag model containing the one previous look yielded a significant improvement in fit over a model containing no previous looks (Empty). Model-building continued iteratively. For example, the Lag 2 model with two previous looks yielded a significant improvement in fit over a model with one previous look. The Lag 6 model including one through six previous looks back fit significantly better than the Lag 5 model containing one through five previous looks. However, fixed effects parameters for two of the six previous looks were not significant, and this model was not interpreted, resulting in a 5 lag model (see Table 3). The inclusion of a Lag 1 Duration as predictor accounted for a 16.0% increase in variance accounted for compared to a model with the intercept alone. Subsequent lags increased variance accounted for by the following compared to each previous lag as follows: Lag 2 – 3.7%, Lag 3 – 6.1%, Lag 4 – 4.7%, Lag 5 – 1.9%, Lag 6 – 3.2%. All models employed an unstructured variance/covariance matrix (see Table S2 for equation and matrix of the 5 lag model). |
| --- |
