# Supplementary material for: Temporal Dependency and the Structure of Early Looking
Source: PLoS One. 2017 Jan 11;12(1):e0169458. doi: 10.1371/journal.pone.0169458 (PMC5226676; doi:10.1371/journal.pone.0169458)
Supplement: S2 Table — (DOCX) [file pone.0169458.s003.docx]

| S2 Table. *5-Lag Model Covariances* | | | | | | |
| --- | --- | --- | --- | --- | --- | --- |
|  | *Covariances (SE)* | | | | | |
|  | *Intercept* | *Lag 1* | *Lag 2* | *Lag 3* | *Lag 4* |  |
| *Lag 1 Duration* | -.003 (.003) |  |  |  |  |  |
| *Lag 2 Duration* | .003 (.003) | -.000 (.004) |  |  |  |  |
| *Lag 3 Duration* | .003 (.003) | -.006 (.005) | -.003 (.004) |  |  |  |
| *Lag 4 Duration* | -.000 (.003) | -.005 (.005) | -.004 (.004) | .000 (.004) |  |  |
| *Lag 5 Duration* | -.005 (.003 | .004 (.004) | -.001 (.003) | -.002 (.004) | .001 (.004) |  |
| *Note:* The 5 Lag model is parameterized in the following equation: ${Look}_{ij}=\beta_{00}+\beta_{10}{Look}_{n-1 ij}+\beta_{20}{Look}_{n-2 ij}+\beta_{30}{Look}_{n-3 ij}+\beta_{40}{Look}_{n-4 ij}+\beta_{50}{Look}_{n-5 ij}+(\varepsilon_{ij}+r_{0i}+r_{1i}{Look}_{n-1 ij}+r_{2i}{Look}_{n-2 ij}+r_{3i}{Look}_{n-3 ij}+r_{4i}{Look}_{n-4 ij}+r_{5i}{Look}_{n-5 ij}+r_{0i}*r_{1i}{Look}_{n-1 ij}+r_{0i}*r_{2i}{Look}_{n-2 ij}+r_{0i}*r_{3i}{Look}_{n-3 ij}+r_{0i}*r_{4i}{Look}_{n-4 ij}+r_{0i}*r_{5i}{Look}_{n-5 ij}+r_{1i}{Look}_{n-1 ij}*r_{2i}{Look}_{n-2 ij}+r_{1i}{Look}_{n-1 ij}*r_{3i}{Look}_{n-3 ij}+r_{1i}{Look}_{n-1 ij}*r_{4i}{Look}_{n-4 ij}+r_{1i}{Look}_{n-1 ij}*r_{5i}{Look}_{n-5 ij}+r_{2i}{Look}_{n-2 ij}*r_{3i}{Look}_{n-3 ij}+r_{2i}{Look}_{n-2 ij}*r_{4i}{Look}_{n-4 ij}+r_{2i}{Look}_{n-2 ij}*r_{5i}{Look}_{n-5 ij}+r_{3i}{Look}_{n-3 ij}*r_{4i}{Look}_{n-4 ij}+r_{3i}{Look}_{n-3 ij}*r_{5i}{Look}_{n-5 ij}+r_{4i}{Look}_{n-4 ij}*r_{5i}{Look}_{n-5 ij})$. | | | | | |  |
