# Supplementary material for: Temporal Dependency and the Structure of Early Looking
Source: PLoS One. 2017 Jan 11;12(1):e0169458. doi: 10.1371/journal.pone.0169458 (PMC5226676; doi:10.1371/journal.pone.0169458)
Supplement: S3 Table — (DOCX) [file pone.0169458.s004.docx]

| S3 Table. *Comparison of Alternate Habituation Models* | | | | | |
| --- | --- | --- | --- | --- | --- |
| Model | Deviance | Number of Parameters | Level 1 Observations (Individual Looks) | AIC | BIC |
| Log10 Look Count | 2647.41 | 6 | 3536 | 2659.41 | 2668.70 |
| Inverse of Look Count | 2930.60 | 6 | 3536 | 2942.60 | 2951.89 |
| First Look of a Trial | 3195.90 | 6 | 3536 | 3207.90 | 3217.19 |
| Untransformed Look Count | 2844.11 | 6 | 3536 | 2856.11 | 2865.40 |
| Untransformed Look Count and Look Count Squared | 2707.91 | 10 | 3536 | 2727.91 | 2743.40 |
| Count of Looks within Trial | 3261.50 | 6 | 3536 | 3273.50 | 3282.80 |
| Trial Number | 2902.64 | 6 | 3536 | 2914.64 | 2923.93 |
| Cumulative Length of Looks | 2897.76 | 6 | 3536 | 2909.76 | 2919.05 |
| Cumulative Length of Looks and  Length of Looks Squared | 2887.58 | 10 | 3536 | 2907.58 | 2923.06 |
| Note: Alternate habituation models are compared. The Log10 Look Count model had the lowest AIC and BIC values. Consequently Log10 Look Count was used to model habituation. Each compared model contained fixed effects for the intercept and the habituation term or terms listed in the first column, as well as their variances, and all possible covariances. For example, the equation for the Log10 Look Count model is as follows:${Look}_{ij}=\beta_{00}+\beta_{10}{Log10 Look Count}_{ij}+(\varepsilon_{ij}+r_{0i}+r_{1i}{Log10 Look Count}_{ij}+r_{0i}*r_{1i}{Log10 Look Count}_{ij})$*.* The covariance (*SE*) for the Log10 Look Count model was: $\tau_{01}\left( r_{0},r_{1} \right)=-.010 \left( .005 \right).$ The inclusion of Log10 Look Count as predictor accounted for a 27.0% increase in variance accounted for compared to a model with the intercept alone. | | | | | |
