# Supplementary material for: Temporal Dependency and the Structure of Early Looking
Source: PLoS One. 2017 Jan 11;12(1):e0169458. doi: 10.1371/journal.pone.0169458 (PMC5226676; doi:10.1371/journal.pone.0169458)
Supplement: S1 Text — (DOCX) [file pone.0169458.s005.docx]

**S1 Text. Model Information**

**Final Model Predicting Look Durations (Manuscript Table 4)**

The final model predicting look durations employed the following equation: ${Look}_{ij}=\beta_{00}+\beta_{10}{Look}_{n-1 ij}+\beta_{20}{Log10 Look Count}_{ij}+(\varepsilon_{ij}+r_{0i}+r_{1i}{Look}_{n-1 ij}+r_{2i}{Log10 Look Count}_{ij}+r_{0i}*r_{1i}{Look}_{n-1 ij}+r_{0i}*r_{2i}{Log10 Look Count}_{ij}+r_{1i}{Look}_{n-1 ij}*r_{2i}{Log10 Look Count}_{ij})$. The final model had an unstructured variance/covariance matrix. In addition to the reported variances, the covariances (*SE*) for the final model were: $\tau_{01}\left( r_{0},r_{1} \right)=-.006 \left( .003 \right), \tau_{02}\left( r_{0},r_{2} \right)=-.020 \left( .006 \right), \tau_{12}\left( r_{1},r_{2} \right)=.002 \left( .007 \right).$

**Final Model Predicting the First Individual Look of a Habituation Trial (Manuscript Table 5)**

The final model predicting the first look of a habituation trial employed the following equation:${First Look}_{ij}=\beta_{00}+\beta_{10}{Look}_{n-1 ij}+\beta_{20}{Log10 Look Count}_{ij}+(\varepsilon_{ij}+r_{0i}+r_{1i}{Look}_{n-1 ij}+r_{2i}{Log10 Look Count}_{ij}+r_{0i}*r_{1i}{Look}_{n-1 ij}+r_{0i}*r_{2i}{Log10 Look Count}_{ij}+r_{1i}{Look}_{n-1 ij}*r_{2i}{Log10 Look Count}_{ij})$. The final model had an unstructured variance/covariance matrix. In addition to the reported variances, the covariances (*SE*) for the final model were: $\tau_{01}\left( r_{0},r_{1} \right)=-.001 \left( .005 \right), \tau_{02}\left( r_{0},r_{2} \right)=-.016 \left( .008 \right), \tau_{12}\left( r_{1},r_{2} \right)=.002 \left( .014 \right).$

**Final Model Predicting Trial Durations (Manuscript Table 6)**

The final model predicting trial durations employed the following equation: ${Trial}_{ij}=\beta_{00}+\beta_{10}{Trial}_{n-1 ij}+\beta_{20}{Log10 Trial Count}_{ij}+(\varepsilon_{ij}+r_{0i}+r_{1i}{Trial}_{n-1 ij}+r_{2i}{Log10 Trial Count}_{ij}+r_{0i}*r_{1i}{Trial}_{n-1 ij}+r_{0i}*r_{2i}{Log10 Trial Count}_{ij}+r_{1i}{Trial}_{n-1 ij}*r_{2i}{Log10 Trial Count}_{ij}). The final model had an unstructured variance/covariance matrix. In addition to the reported variances, the covariances (SE) for the final model were: \tau_{01}\left( r_{0},r_{1} \right)=-.020 \left( .012 \right), \tau_{02}\left( r_{0},r_{2} \right)=.010 \left( .007 \right), \tau_{12}\left( r_{1},r_{2} \right)=.003 \left( .020 \right). \tau_{01}\left( r_{0},r_{1} \right)=-.001 \left( .005 \right), \tau_{02}\left( r_{0},r_{2} \right)=-.016 \left( .008 \right), \tau_{12}\left( r_{1},r_{2} \right)=.002 \left( .014 \right).$
